# Poverty and Child Behavioral Problems: The Mediating Role of Parenting and Parental Well-Being

**DOI:** 10.3390/ijerph14090981

**Published:** 2017-08-30

**Authors:** Till Kaiser, Jianghong Li, Matthias Pollmann-Schult, Anne Y. Song

**Affiliations:** 1 WZB Berlin Social Science Center, 10785 Berlin, Germany; jianghong.li@wzb.eu (J.L.); anne.song@wzb.eu (A.Y.S.); 2 Faculty of Psychology, Ruhr-University Bochum, 44801 Bochum, Germany; 3 Telethon KIDS Institute, the University of Western Australia, Subiaco WA 6008, Australia; 4 Center for Population Health Research, Curtin University, Perth WA 6102, Australia; 5 Faculty of Humanities, Social Science and Education, Magdeburg University, 39106 Magdeburg, Germany; Matthias.Pollmann-Schult@ovgu.de

**Keywords:** poverty, life satisfaction, parenting, child behavior problems, Germany

## Abstract

The detrimental impact of poverty on child behavioral problems is well-established, but the mechanisms that explain this relationship are less well-known. Using data from the Families in Germany Study on parents and their children at ages 9–10 (middle childhood), this study extends previous research by examining whether or not and to what extent different parenting styles and parents’ subjective well-being explain the relationship between poverty and child behavior problems. The results show that certain parenting styles, such as psychological control, as well as mothers’ life satisfaction partially mediate the correlation between poverty and child behavioral problems.

## 1. Introduction

A large body of research has demonstrated that poverty, low income, and low socioeconomic status are linked to behavioral problems in children and adolescents [1,2,3,4]. However, the mechanisms that explain this association remain a major topic in this line of research. Many of the previous studies are guided by the Family Stress Model (FSM) [5,6] or the Family Investment Model (FIM) [7,8]. These models argue, respectively, that family financial difficulties, by negatively impacting emotional and relationship functioning, disrupt effective parenting practices, and the lack of economic resources requires parents to focus on immediate material needs, thus limiting the investment in developmentally supportive conditions for their offspring. Indeed, previous studies have suggested that parental mental health and parenting styles are important mechanisms underpinning the correlation between low income and poverty and child behavior problems. Low income and poverty were linked to inconsistent, unsupportive, and uninvolved parenting styles and poor parental mental health, which in turn are associated with child behavior problems.

For instance, in a recent study based on the UK Millennium Cohort Study, Fitzsimons et al. [2] showed that persistent poverty was associated with peer and conduct problems. They also reported that transitions into poor maternal mental health were associated with decreased child mental health in various domains (emotional, peer, conduct, and hyperactivity). Fathers’ mental health was less important, but paternal mental distress was associated with increased chance of children developing emotional problems by age 11. Similarly, Kiernan and Huerta [9] found that economic deprivation was linked to both externalizing and internalizing problems among children through maternal depression.

In Norway, Bøe and colleagues [10] examined the association between parent-rated family economy and child mental health. Their findings show that poorer family economy was associated with externalizing problems in children through lower parental emotional well-being and negative parenting. Family economy was also associated with internalizing problems in children, both directly and indirectly through low parental emotional well-being and negative parenting. However, family economy was based on perceived economic hardship, and the authors recommended further investigation using objective measures of income.

For Germany, Berger and Spiess [11] analyzed self-reported life satisfaction as a measure of subjective well-being from a broader group of mothers from the Socio-Economic Panel Study (SOEP). The authors found that mothers who were more satisfied with life had children with lower behavioral problems. Measures of general life satisfaction correlate strongly with satisfaction scores for different specific domains in life, as well as with non-self-reported measures of well-being [12]. Thus, life satisfaction scales, while not targeting clinical mental health problems specifically, can be a useful additional tool in capturing how people feel in terms of family, work, and external circumstances that affect their life.

It is important to note, however, that the association between economic hardship and child behavior problems can also be mediated through other pathways than parenting practices. Apart from so-called family-based pathways (e.g., parenting practices, health behavior, and parent–child interactions), community-level pathways (e.g., neighborhood safety, exclusion from and bullying by peer groups, school characteristics, and access to health care) also play a crucial role [4,13]. Indeed, empirical research shows adverse effects of low socioeconomic status (SES) neighborhoods on children’s behavioral and emotional problems when family-level characteristics were taken into account [14]. A great number of studies have also pointed to the effect of social policies on child behavioral problems [15]. Thus, higher levels of behavior problems among poor children cannot be solely attributed to the family’s economic hardship (and accompanying adverse parenting practices). Community-level factors and access to institutional resources do matter. In fact, qualitative evidence for Germany suggests that poor parents are able to parent adequately, despite severe financial constraints [16].

One limitation of previous research is that the researchers did not consider fathers’ parenting or fathers’ reports of children’s emotional and behavioral problems. Fathers play an equally important role in children’s development [17,18,19]. Moreover, while previous research has investigated a specific parenting style as a mechanism, no studies have examined parenting in a comprehensive way. Within the broader context of community-level and institutional determinants of child behavioral problems as discussed above, our study aims to add to the existing research in several ways. We examine the mediating role of five parenting styles and of parental life satisfaction in order to obtain a comprehensive overview of the effect of parenting and parents’ general subjective well-being on child emotional and behavioral problems. In addition, we incorporate information from both mothers and fathers. This is in contrast to most previous studies, which, due to data restrictions, used only mothers’ ratings of child behavior and parenting styles.

## 2. Hypotheses

**Figure 1:**
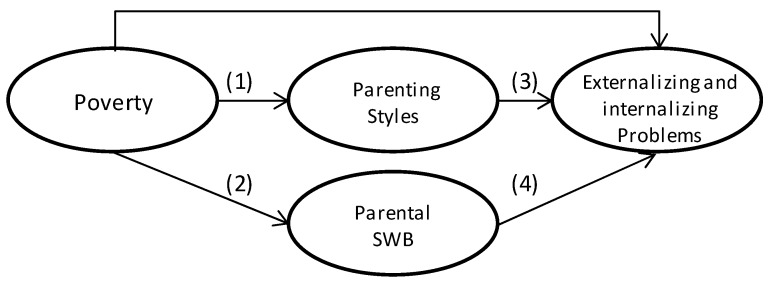
Pathways from poverty to child behavioral problems. SWB: subjective well-being.

Figure 1 summarizes our hypotheses. The numbers correspond to the pathways leading from poverty to child behavioral problems.

- Parenting styles mediate the correlation of poverty with child behavior problems (both internalizing and externalizing) through pathways 1 and 3.
- Mothers’ and fathers’ subjective well-being mediates the correlation between poverty and child behavioral problems (both internalizing and externalizing) through pathways 2 and 4.

## 3. Materials and Methods

### 3.1. Data

The data for this study were derived from the “Families in Germany” Study (FiD) [20], which is an extension of the German Socio-Economic Panel Study (SOEP) [21]. The FiD targets single parents, large families with more than two children, low-income families, and a sample of households with children born between 2007 and 2010 that was randomly sampled from German national registries. The survey started in 2010 and collected information on parents and their children ages 0 to 10. Our analysis was based on both parents’ reports of child behavior problems from all available waves (2010–2013) for children ages 9 to 10 years. Our sample includes only families with two parents, but we also did robustness checks including lone fathers and lone mothers (see sensitivity analysis). We pooled the data from all four waves and our analytical sample included 1097 children from 922 households.

### 3.2. Method

We used structural equation modeling to test our hypotheses. The statistical significance of the mediating (indirect) effects was tested by using the resampling method of bootstrapping with bias-corrected confidence intervals [22]. Because some children were siblings and lived in the same household, we used the robust maximum likelihood estimation to take into account clustered standard errors at the household level. The FiD data offers us the rare opportunity to analyze both fathers’ and mothers’ rating of their parenting style and their children’s behavior problems. For our analysis, we combined these dual ratings into latent variables to increase the reliability of these constructs. We combined the ratings of fathers’ and mothers’ parenting style, since previous research suggests a similar logic of child rearing for both parents (e.g., Lareau, 2011) [23]. The use of latent variables allows for a correction of measurement errors and thus reduces biased estimates [24]. We also estimated the model separately for mothers and fathers, including single parents, to ascertain the robustness of the results (see sensitivity analysis).

We are aware of the cross-sectional nature of our data and therefore we refrain from making causal statements. However, to make it easier to follow our analysis, we used the terms “direct effects”, “indirect effects”, and “total effects” in the description of the results, as they are commonly used in mediation analysis.

### 3.3. Measures

#### 3.3.1. Endogenous Variables

*Children’s behavior problems* were measured with the Strengths and Difficulties Questionnaire (SDQ) which was developed by Goodman [25]. We were able to analyze the child SDQ scale based on fathers’ and mothers’ reports. The SDQ covers four domains: hyperactivity (Mother: α = 0.80; Father: α = 0.78), emotional problems (Mother: α = 0.70; Father: α = 0.69), conduct problems (Mother: α = 0.60; Father: α = 0.69), and peer problems (Mother: α = 0.64; Father: α = 0.61). Each domain includes five items on a scale of (1) *Does not apply* to (3) *Fully applies*. These four domains of SDQ were aggregated into two subscales: externalizing problems and internalizing problems. A higher score corresponds to more behavioral problems. *Externalizing problems* (Mother: α = 0.82.; Father: α = 0.81) include the domains for conduct problems and hyperactivity, while *internalizing problems* (Mother: α = 0.76; Father: α = 0.75) comprise the emotional and peer problems domains.

Mediators: we tested five different *parenting styles* as mediators: inconsistent parenting, strict control, psychological control, negative communication, and emotional warmth. These parenting style scales are each measured with three items on a Likert-scale from 1 (*Never*) to 5 (*Frequently*) and are rated by both mothers and fathers. *Inconsistent parenting* (Mother: α = 0.72; Father: α = 0.70) is based on the expanded German version of the Alabama Parenting Questionnaire (EDAPQ) [26], and consists of the three items: “I reduce punishments or end them early”, “I threaten my child with a punishment but do not actually follow through”, and “I find it hard to set and keep consistent rules for my child”.

The *emotional warmth* scale (Mother: α = 0.69; Father: α = 0.75) is based on the questionnaires developed by Perris et al. [27] and Schumacher et al. [28]. It includes the items: “I show my child with words and gestures that I care about him/her”, “I console my child when he/she is sad”, “I praise my child”. The *negative communication* scale (Mother: α = 0.57; Father: α = 0.64) is based on an instrument developed by Schwarz et al. [29], and includes: “I criticize my child”, “I yell at my child when he/she does something wrong”, “I scold my child when I am angry at him/her”. The same is true for the scale *strict control* (Mother: α = 0.48; Father: α = 0.53), which includes the items: “I tend to be a strict parent”, “If my child does something against my will, I punish him/her”, “I make it clear to my child that he/she is not to break my rules or question my decisions”. *Psychological control* (Mother: α = 0.52; Father: α = 0.51) is based on the Zurich Brief Questionnaire for the Assessment of Parental Behaviours [30] and consists of the items: “I am disappointed and sad when my child misbehaves”, “I think my child is ungrateful when he/she does not obey me”, “I do not talk to my child for a while when he/she does something wrong”.

Parental *life satisfaction* (as an indicator of parental well-being) is measured with one item (“How satisfied are you with your life, all things considered?”) on a scale of 0 (*totally unsatisfied*) to 10 (*totally satisfied*).

#### 3.3.2. Exogenous Variables

The main exogenous variable is *poverty* (1 = Poor, 0 = Not poor). We used the measure of relative poverty defined by the OECD: 60% of the median equivalent household income [31]. The median was estimated using data from the German Socio-Economic Panel Study (SOEP). We controlled for child sex and age in months, the number of children in the household, mothers’ age, child care attendance, whether or not one or both parents had a migration background, and region (East vs West Germany). Table 1 provides an overview of all variables analyzed.

## 4. Results

**Figure 2:**
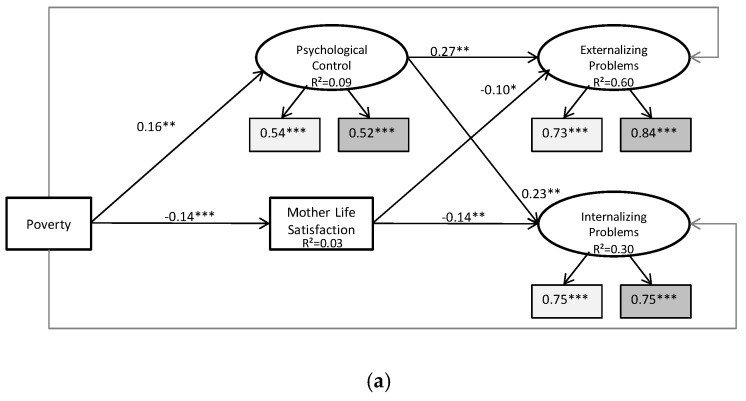

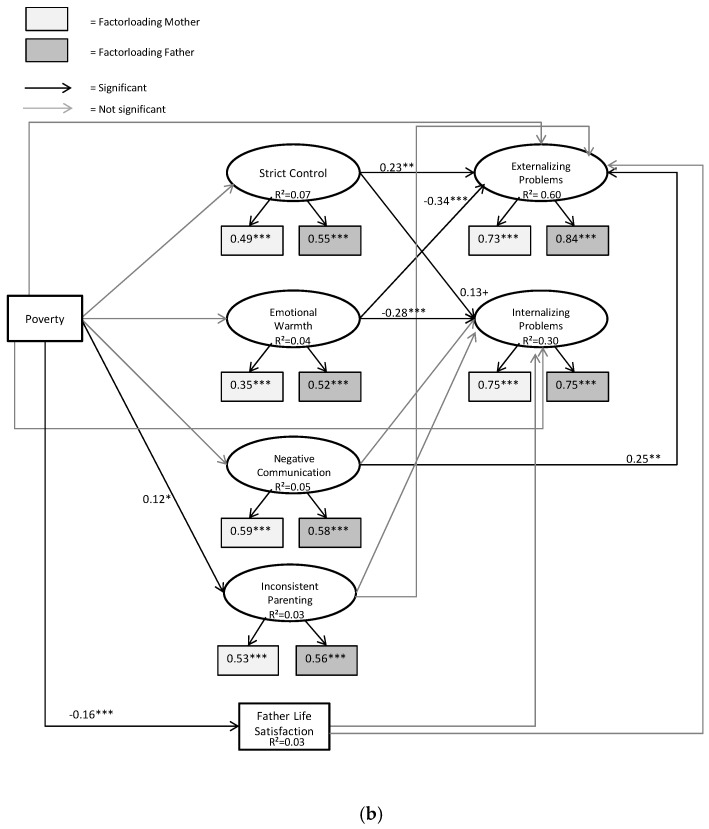
Poverty and child behavioral problems: (**a**) The mediating role of psychological control and mothers’ life satisfaction; (**b**) The mediating role of other parenting styles and fathers’ life satisfaction. Figure includes only significant mediators. *N* = 1097; control variables: sex, age (child and mother), migration background, number of children in the household, sample-region, and child care attendance. Model fit: chi ² = 357.407, df (125), *p* < 0.000, CFI 0.94, TLI 0.88, RMSEA 0.04, and SRMR 0.04. Tables including unstandardized coefficients, factor loadings, and residual covariances/correlations are available upon request. Levels of significance: *** *p* < 0.001, ** *p* < 0.01, * *p* < 0.05, + *p* < 0.1.

The results from the structural equation model (Figure 2a,b) show no significant direct effect of poverty on internalizing (β = 0.03, *p* = 0.509) and externalizing problems (β = 0.04, *p* = 0.429). However, this does not mean that poverty is not linked to child behavior problems: it is possible that the effect of poverty on child behavior problems is fully mediated, and this becomes evident in Figure 2a. We found significant direct effects of poverty on psychological control (β = 0.16, *p* < 0.01), inconsistent parenting (β = 0.12, *p* < 0.05), and mothers’ life satisfaction (β = −0.14, *p* < 0.001). Furthermore, psychological control (β = 0.23, *p* < 0.01) and mothers’ life satisfaction (β= –14, *p* < 0.01) have significant direct effects on internalizing problems. This is also true for externalizing problems (psychological control: β = 0.27, *p* < 0.01; mothers’ life satisfaction β = −0.10, *p* < 0.05). This suggests that full mediation may indeed occur.

Although poverty has significant direct effects on inconsistent parenting (β = 0.12, *p* < 0.05) and fathers’ life satisfaction (β = −0.16, *p* < 0.001), neither are significant predictors of child behavior problems.

To formally test for mechanisms, we calculated the indirect effects (see Table 2). Although there is no direct effect of poverty on child behavior problems, there are indirect effects through parenting styles. We observe from Table 2 that the largest part of the effect of poverty on overall child behavior problems is explained by psychological control. For externalizing problems, about one-third of the total effect of poverty is explained by psychological control (β = 0.04, *p* < 0.05), and this also holds true for internalizing behaviors (β = 0.04, *p* < 0.10). Mothers’ life satisfaction also plays a mediating role: poverty has a significant indirect effect on internalizing problems via mothers’ life satisfaction(β = 0.02, *p* < 0.01). The same holds true for externalizing problems, although with marginal statistical significance (β = 0.01 *p* < 0.10).

As shown in Figure 2b, the other parenting styles and fathers’ life satisfaction do not play a mediating role in the relationship between poverty and child behavioral problems. Although emotional warmth and strict control are significantly associated with child behavioral problems, they do not mediate the effect of poverty on neither internalizing nor externalizing problems.

### Sensitivity Analysis

In additional analyses, we estimated our model separately for fathers and mothers. These analyses produced findings similar to that in our main model. The only difference was that we found additional mediation effects via fathers’ life satisfaction in the fathers model and mediation effects of inconsistent parenting in the mothers model. Our main model does not include lone parents; therefore, we tested whether lone parents systematically differed in their parenting styles by conducting t-tests. While for fathers we did not find significant differences, we found small, but statistically significant differences among mothers with regard to two parenting styles: “emotional warmth” (means: lone mothers = 4.54; partnered mothers = 4.48) and “strict control” (means: lone mothers = 2.84; partnered mothers = 2.92). However, neither of the parenting styles played a role as a mediator.

For additional robustness checks, we estimated our model using sampling weights that are provided by the FiD study. Estimating the model with weighted data, however, caused convergence problems, which may be due to the high complexity of the model. The use of a more parsimonious model that only tested the significant mediation pathways produced similar results to those from our main model.

## 5. Discussion

The findings support our hypotheses. Our results show that the effect of poverty on child behavior problems is partially mediated through parenting styles (psychological control) as well as maternal subjective well-being (mothers’ life satisfaction). While poverty lowers fathers’ life satisfaction, the latter is not associated with child behavioral problems. This may be attributed to the fact that, in Germany, the male breadwinner model still prevails and therefore fathers spend more time at work and less time with children in the home than mothers do. Even when mothers are gainfully employed (either part time or full time), they not only retain a larger share of actual household work (e.g., washing, cooking, and cleaning) but also do most of the planning for and thinking about the immediate and future needs of the family and children. This latter part of the household responsibility is what some feminists call “mental load” or “emotional work” for mothers, which may make mothers’ life satisfaction more connected to children’s behavioral development. The strict control and emotional warmth parenting styles were linked to internalizing and externalizing behaviors, but they did not explain the relationship between poverty and child behavioral problems. The other two parenting styles (negative communication and inconsistent parenting) were not associated with child behavioral problems in our study sample. Psychological control stood out as the only parenting style that mediated in part the relationship between poverty and child behavioral problems, possibly because it may represent the harshest parenting behavior among all five parenting styles, and hence its impact on child behavioral problems may be more enduring (e.g., parents do not talk to the child for a while when he/she does something wrong) compared to other parenting styles.

Our study contributes to the literature by incorporating fathers’ perspectives of child behavioral problems and their parenting as an equally important mediating factor underpinning the link between poverty and child emotional and behavioral problems. This can be observed in the similar factor loading for both maternal and paternal responses to the items on psychological control and on child internalizing and externalizing problems. Our findings demonstrate that out of all of the parenting styles, psychological control plays an important role in understanding how poverty negatively impacts on child behavior. The study further shows that it is not only parental mental health problems, such as depression or distress [2,9,10], that play a role in the link between poverty and child behavioral problems, but also that parental general well-being, such as mothers’ satisfaction with life, is important for understanding this link. One major limitation of our study is that the analysis was based on cross-sectional data, and hence we cannot make any causal inferences from our findings. Further research using longitudinal data is warranted to elucidate the causal pathways from poverty and economic deprivation to child behavioral problems involving harsh parenting styles and parental subjective well-being.

## 6. Conclusions

Our finding that both mothers’ and fathers’ parenting styles play an import role in understanding the influence of poverty on child behavioral problems has policy implications. At the upstream level, policy interventions ought to tackle the causes of poor parenting styles in low income families. Research suggests that work stress is associated with poor parenting styles (coercive or permissive parenting styles) [32,33]. Parents from low income families are confronted with multiple stressors and challenges, such as financial stress, work-related stress (working multiple jobs, poor working conditions and non-standard work schedules), and coping with their physical and mental health problems with lack of or limited access to health care services. All of these stressors may contribute to poor parenting practices. An improvement in the provision of social and economic support and health care is likely to reduce poor parenting practice in low income families. At the intermediate level, a parenting program, such as the Triple P-Positive Parenting Program [34], tailored for poor families would be also effective for alleviating the detrimental effect of poverty on child behavioral problems.

## Figures and Tables

**Table 1:** Frequency distribution of all variables (*N* = 1097).

Variables	Mean/Percent	SD	Range	Mean/Percent	SD	Range
	Mother			Father		
SDQ						
Internalizing problems	3.94	3.50	0–18	3.82	3.30	0–19
Externalizing problems	5.17	3.79	0–18	5.69	3.64	0–20
Poverty						
Poverty = yes	18.41%	-	0–1			
Parenting styles						
Psychological control	3.75	0.50	1.5–5	1.95	0.59	1–5
Strict control	2.50	0.57	1–4.3	2.93	0.61	1–5
Emotional warmth	4.48	0.47	1.7–5	4.04	0.59	1–5
Inconsistent parenting	2.57	0.72	1–5	2.60	0.72	1–4.67
Negative communication	2.50	0.57	1–4.3	2.47	0.60	1–5
Life satisfaction	7.77	1.62	0–10	7.61	1.58	0–10
Household variables						
Maternal age	39.16	5.23	25–58			
Migration background = yes	33.55%	-	0–1			
East Germany = yes	15.13%		0–1			
Child care = yes	33.09%		0–1			
Child sex = female	51.69%	-	0–1			
Child age (in month)	118.38	3.62	110–127			
Number of children	3.08	1.05	1–4.67			

SDQ: Strengths and Difficulties Questionnaire.

**Table 2:** Effect decomposition.

Poverty	Strengths and Difficulties	
	Externalizing Problems	Internalizing Problems
Direct effect	0.04	0.03
Indirect effects		
Parenting styles		
via psychological control	0.04 *	0.04 ^+^
via inconsistent parenting	0.01	0.01
via strict control	−0.00	−0.00
via emotional warmth	0.03	0.03
via inconsistent parenting	0.01	0.01
via negative communication	−0.00	−0.00
Life satisfaction		
Mother	0.01 ^+^	0.02 **
Father	0.01	0.01
Total effect	0.14 ***	0.12 **

*** *p* < 0.001, ** *p* < 0.01, * *p* < 0.05, ^+^ *p* < 0.1.
